# Older adults in jail: high rates and early onset of geriatric conditions

**DOI:** 10.1186/s40352-018-0062-9

**Published:** 2018-02-17

**Authors:** Meredith Greene, Cyrus Ahalt, Irena Stijacic-Cenzer, Lia Metzger, Brie Williams

**Affiliations:** Department of Medicine, Division of Geriatrics, University of California San Francisco, San Francisco, CA USA

**Keywords:** Criminal justice, Jail, Geriatric conditions

## Abstract

**Background:**

The number of older adults in the criminal justice system is rapidly increasing. While this population is thought to experience an early onset of aging-related health conditions (“accelerated aging”), studies have not directly compared rates of geriatric conditions in this population to those found in the general population. The aims of this study were to compare the burden of geriatric conditions among older adults in jail to rates found in an age-matched nationally representative sample of community dwelling older adults.

**Methods:**

This cross sectional study compared 238 older jail inmates age 55 or older to 6871 older adults in the national Health and Retirement Study (HRS). We used an age-adjusted analysis, accounting for the difference in age distributions between the two groups, to compare sociodemographics, chronic conditions, and geriatric conditions (functional, sensory, and mobility impairment). A second age-adjusted analysis compared those in jail to HRS participants in the lowest quintile of wealth.

**Results:**

All geriatric conditions were significantly more common in jail-based participants than in HRS participants overall and HRS participants in the lowest quintile of net worth. Jail-based participants (average age of 59) experienced four out of six geriatric conditions at rates similar to those found in HRS participants age 75 or older.

**Conclusions:**

Geriatric conditions are prevalent in older adults in jail at significantly younger ages than non-incarcerated older adults suggesting that geriatric assessment and geriatric-focused care are needed for older adults cycling through jail in their 50s and that correctional clinicians require knowledge about geriatric assessment and care.

## Background

The proportion of U.S. citizens who are incarcerated in prison or jail is higher than any other western nation (Walmsley 2000). Every year, over 11 million persons are admitted to U.S. jails (Minton and Zeng 2015) of whom a rapidly growing number are older adults. From 1996 to 2008, the number of older adults in jail who were age 55 or older increased 278% compared to a 53% increase in the overall jail population (Beck and Berzofsky 2010; Darrell and Beck 1997) and an estimated 500,000 older adults are arrested and pass through jails each year (Snyder 2012). Life experiences that confer risk of poor future health are disproportionately common among justice-involved adults, such as limited access to quality healthcare and education over the lifespan; a history of substance use disorder or homelessness; and experiences of significant stress or trauma (Williams et al. 2012a; Maschi et al. 2011; Bedard et al. 2017). In addition, chronic health conditions are disproportionately common among the incarcerated (Williams et al. 2012a; Maschi et al. 2011; Bedard et al. 2017). The cumulative burden of multiple chronic conditions and adverse health risks has led some criminal justice researchers and professionals to contend that prisoners may experience “*premature*” or “*accelerated*” aging (Aday 2003; Williams et al. 2012b). As a result, the age at which criminal justice-involved individuals are considered “older” varies considerably, with some systems using the community norm (age 65) and others using ages 50 or 55 to define “older age” (Psick et al. 2017).

While several studies have compared chronic health conditions between incarcerated and non-incarcerated individuals (Binswanger et al. 2009) chronic conditions alone are not the best judge of health in older adults. Instead, health needs and health status in older age are better described according to the presence of geriatric conditions (e.g., limitations in the ability to complete one’s Activities of Daily Living (ADLs – eating, bathing, transferring, toileting or dressing), mobility impairment, falls, incontinence, multimorbidity) (Covinsky et al. 1997; Covinsky et al. 2000). Yet no study to our knowledge has compared rates of geriatric conditions in incarcerated older adults to a population-based sample of community-dwelling older adults. As a result, it is unclear whether the aging-related healthcare needs of this population differ from older adults in the community.

Focusing exclusively on chronic disease management without consideration of geriatric conditions risks doing little to develop and target healthcare models for this population that improve health-related quality of life, address increased risk of mortality (Binswanger et al. 2007) and acute care use (Chodos et al. 2014) and lower high costs of care (Ahalt et al. 2013). Furthermore, a clearer understanding about the comparative prevalence of geriatric conditions according to age in this population could help to determine whether the concept of “accelerated aging” is appropriate for this population. Therefore, we conducted a cross-sectional study to describe the burden of geriatric conditions according to age in older adults in jail, a medically vulnerable population that is often under-represented in national health surveys, and to compare these rates to an age-matched sample of community dwelling non-incarcerated older adults represented in a commonly used national survey (the Health and Retirement Study) (Juster 1995). The goal of this study is to generate the knowledge needed by health and criminal justice systems and professionals tasked with meeting the health and health-related social service needs of this growing population. Given the risk factors for poor health that are common in this population, we hypothesized that older adults in jail would have higher rates of geriatric conditions than the general population consistent with the assumed phenomenon of “accelerated aging.”

## Methods

### Design and participants

#### Jail-based study

A cross-sectional study conducted between May 15 and November 15, 2012 consecutively enrolled 238 older adults age 55 or older who spent at least 48 h in an urban county jail. The 48-h cut-off was used because incarcerated individuals may be in transit or in court in the time period shortly after arrest and unable to participate in a research study. Eligible participants spoke English, Spanish or Cantonese, and were deemed not to be a safety risk by the Sheriff’s deputy on duty. Participants were enrolled in the study using a consecutive sampling approach. Individuals who met our criteria and were available during business hours were contacted by a nurse in the jail to determine their interest in hearing more about the study. Research staff only approached those individuals who agreed to be contacted. Enrollment of all eligible, available, and interested participants occurred over a period of 5 months so as to limit the impact of frequent population turnover and seasonal fluctuation in arrest rates on our sample (Hulley et al. 2007). For those who wished to participate, consent was obtained using a teach-to-goal method that enables research staff to evaluate participants’ understanding of consent forms before enrolling them in the study (Sudore et al. 2006). In teach-to-goal, consent forms are written at a fifth grade reading level and are also read out loud to participants, who are given unrestricted time to review the forms with trained staff. Potential participants are then asked to describe the research procedures or to answer questions about the study. Misperceptions are corrected and the participant’s comprehension is assessed again. Those who cannot demonstrate comprehension after several attempts are excluded from the study (Ahalt et al. 2017).

Study questionnaires were read to participants and answers were transcribed by the interviewers. Jail medical records were abstracted for participants who granted access. Consistent with federal and state regulations governing human subjects research involving prisoners, (Hanson et al. 2012) $10 was deposited in participants’ jail accounts to compensate them for their time. Due to the small number of women enrolled in this study (*n* = 12) and because 86% of jail inmates nationwide are male (Minton and Zeng 2015) only men were included in these analyses.

#### Community-based study

Jail-based participants were compared to those in a national sample of community-dwelling older adults in the Health and Retirement Study (HRS – 2012 study wave). The HRS is a longitudinal population-based study of community dwelling adults aged 50 or older which selects participants based on a nationally representative multistage probability sample of households (Juster 1995). HRS uses standard measures to collect self-reported health and sociodemographic information. For this study, HRS participants were restricted to males aged 55 or older (to mirror the gender and age cutoffs for jail participants). Only participants who did not require a proxy respondent were included since all jail-based participants had to be able to provide their own informed consent. Study participants in HRS were specifically chosen as a comparison group given its focus on older adults, inclusion of geriatric condition outcomes, and overall similarity of study measures for comparison purposes.

### Measures

#### Sociodemographics

The same sociodemographic measures (age, race/ethnicity, education, gender) were used in both studies. Annual income among jail-based participants was categorized as $15,000 or less based on the Medicaid minimum income eligibility cut-off. HRS measures household net worth for each participant. Net worth, a comprehensive assessment of all assets and debts, is considered to be the gold standard measurement for socioeconomic status in older adults (Pollack et al. 2007). We categorized participants in HRS according to net worth quintiles (lowest net worth quintile <$24,000). The same measures were used in each study for recent heavy drinking (drinking 5 or more drinks on a typical day, an item from the validated Alcohol Use Disorders Identification Test [AUDIT-C] brief screening test for problem drinking) (Bush et al. 1998) and current tobacco use (“do you smoke cigarettes?”). Homelessness (defined as needing to spend one or more nights outside or in a homeless shelter in the 30 days prior to jail) (Marquez 2011) and drug use (“No” to “In the last year, could you get through the week without using drugs?”) (Skinner 1982) were assessed in the jail study but not in HRS.

#### Health conditions

Each study assessed self-rated health (Poor/Fair, Good, or Very Good/Excellent) (Ware et al. 1996). and used the same validated measures of self-report of chronic conditions and depression (Juster 1995). Jail participants also answered questions about health conditions that were not available in the HRS, specifically HIV, Hepatitis C, and serious mental illness (based on the definition used by the Bureau of Justice and Statistics (James and Glaze 2006). Medical charts were reviewed for medical diagnoses for all jail study participants who granted access to their medical record (93%). Although self-report of medical conditions is well-validated in older adults (Bush et al. 1989) and vulnerable populations, (Hwang et al. 2016) this combined approach was taken to minimize underrepresentation of medical diagnoses for jail-based participants who are not aware of their medical conditions.

#### Geriatric conditions

Geriatric conditions (functional, mobility or hearing impairment; multimorbidity; urinary incontinence; and falls) were assessed in each study using identical questions. Functional impairment was defined as having difficulty with one or more Activities of Daily Living [ADLs - bathing, dressing, feeding, toileting or transferring]); mobility impairment (difficulty walking several blocks); hearing impairment (a response of “poor” or “fair” to “Is your hearing excellent, very good, good, fair or poor?”). We defined multimorbidity as the presence of two or more medical conditions (hypertension, diabetes, cancer, lung disease, heart disease, stroke, arthritis, and HIV and Hepatitis C among jail inmates). Falls and urinary incontinence (“have you lost any amount of urine beyond your control”) were assessed over the past 3 months in the jail study and over the past 2 years for HRS participants. Shorter timeframes in the jail study were used to limit false positive reporting while still providing a measure for comparison.

### Statistical analysis

We compared sociodemographics, health status and geriatric conditions using descriptive statistics. All characteristics were compared with an age-adjusted analysis using logistic regression models to account for the difference in age distributions between the two participant groups. We also conducted a second analysis comparing participants in the jail study to HRS participants in the lowest quintile of net worth (<$24,000) to examine the extent to which low-income/low socioeconomic status may account for differences between the two groups. Finally, we compared the burden of geriatric conditions present in each study population according to age strata to determine whether the older adults in jail experienced geriatric conditions at younger ages than their community-dwelling counterparts. All analyses were performed using Stata Version 12 (Stata Statistical Software [computer program] 2011) and all data were managed using REDCap electronic data capture (Harris et al. 2009). The study was approved by the Institutional Review Board at University of California San Francisco.

## Results

Of 319 adults aged 55 years or older who entered jail over the study period, 23 (7%) did not meet inclusion criteria (7 (2%) did not speak English, Spanish, or Cantonese, and 16 (5%) were deemed a safety risk to interviewers). Of the remaining 296, 44 (15%) declined to participate and 252 (85%) were enrolled. Two (< 1%) withdrew and 12 (5%) were women, resulting in a final sample of 238 older male jail-based participants. Of the 238 participants, five interviews were conducted in Spanish and two in Cantonese. Most participants (230, 93%) consented to having their medical chart reviewed. Those who did not meet inclusion criteria or declined to participate did not differ statistically in age from participants. The comparison group included 6871 community-based HRS male participants age 55 or older, of whom 1586 were in the lowest quintile of net worth.

### Sociodemographics and health conditions

Jail-based participants had a mean age of 59 years (range 55 to 75) while HRS participants had a mean age of 67 (range 55 to 100) and HRS participants in the lowest quintile of net worth had a mean age of 64. In age-adjusted analyses, jail-based participants were more likely than both HRS populations (overall and those in the lowest quintile of net worth) to be non-white (80% jail participants, 25% all HRS, 43% lowest quintile of net worth in HRS, all comparisons with jail participants *p* < 0.001), to have less than a high school education (75% vs. 10% vs. 20%, *p* < 0.001) and were less likely to be married (12% vs. 74% vs. 55%, p < 0.001), Table 1. Jail-based participants were more likely than both HRS groups to report being a heavy drinker (17% vs. 6% HRS overall vs. 7% HRS lowest quintile of net worth, p < 0.001) and a current smoker (65% vs. 21% vs. 33%, *p* < 0.001). Rates of hypertension, chronic lung disease, stroke and arthritis were higher among jail-based participants than in the overall HRS cohort (all *p* < 0.05, Table 1) but were statistically similar to rates found in HRS participants from the lowest quintile of net worth (Table 1). Jail-based participants also experienced high rates of Hepatitis C (50%), HIV (12%), serious mental illness (56%), homelessness (45%), and recent drug use (64%). These conditions were not assessed by HRS*.*

**Table 1:** Age-adjusted comparison of older adults in jail with older adults in the Health and Retirement Study (HRS) overall and with older adults in the Health and Retirement Study (HRS) in the lowest net worth quintile (net worth <$24,000)

	Older Adults in Jail (*n* = 238)	HRS overall (*n* = 6871)	HRS in lowest net worth quintile (*n* = 1586)	*P*-Value Jail vs. HRS overall	*P*-Value Jail vs. HRS in lowest net worth quintile
Race/ethnicity^a^					
Non-White Race	79.8%	24.5%	43.0%	< 0.001	< 0.001
Education Level					
< High school	74.8%	10%	21.2%	< 0.001	< 0.001
US Military Veteran	25.2%	27.3%	28.7%	0.501	0.247
Marital Status					
Currently Married	12.2%	73.9%	55.3%	< 0.001	< 0.001
Separated/Divorced/Widowed	54.6%	17.5%	29.6%	< 0.001	< 0.001
Never Married	32.8%	8.8%	15.2%	< 0.001	< 0.001
Self-rated health					
Poor /Fair	53%	22.5%	39.7%	< 0.001	0.009
Good	24.8%	31.6%	31.9%	< 0.001	0.009
Very Good / Excellent	22.2%	45.9%	28.4%	< 0.001	0.009
Hypertension^b^	64.3%	54.3%	63.2%	< 0.001	0.607
Diabetes	18.5%	21.5%	27.2%	0.237	< 0.001
Cancer^c^	7.1%	9.0%	9.0%	0.280	0.258
Chronic lung disease	16.4%	7.2%	12.1%	< 0.001	0.069
Cardiovascular disease^d^	21.8%	19.2%	21.2%	0.329	0.823
Stroke	10.5%	4.9%	8.6%	0.005	0.332
Arthritis	50.4%	43.1%	45.6%	0.020	0.125
Depression (self-report)	27.3%	19.2%	28.0%	0.004	0.887
Serious Mental Illness^e^	55.5%	NA	NA	–	–
HIV	5.1%	NA	NA	–	–
Hepatitis C	49.6%	NA	NA	–	–
Heavy drinker^f^	17.4%	6.3%	6.8%	< 0.001	< 0.001
Current smoker^g^	64.7%	20.7%	32.9%	< 0.001	< 0.001

^a^Further details on race/ethnicity among older adults in jail: White 20%, Black 60%, Latino 9%, other 8%; among overall HRS: White 78%, Black 12%, Latino 9%, Other 3% (*p* < 0.001)

^b^all comorbidities including depression based on combination of self-report and review of jail medical record when available

^c^excludes minor skin cancer

^d^includes history of myocardial infarction, coronary heart disease, angina, congestive heart failure

^e^based on the Bureau of Justice Statistics’ definition of “Serious Mental Illness,” which includes having a diagnosis of major depressive disorder, mania, or a psychotic disorder

^f^defined as having 5 or more drinks on a typical day

^g^response of yes to “Do you smoke cigarettes?”

### Geriatric conditions

**Fig. 1:**
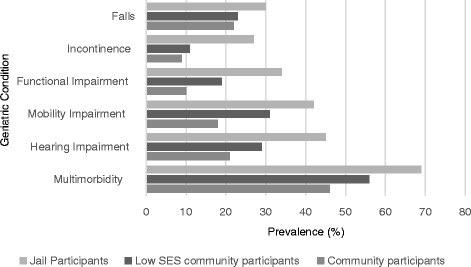
Geriatric conditions in jail cohort vs. HRS population vs lowest net worth quintile HRS population. Older jail-based participants have higher rates of each of six geriatric conditions when compared to age-adjusted community-dwelling populations of older adults in the Health and Retirement Study (HRS) overall and to the subpopulation in the HRS sample in the lowest net worth quintile (*p*-value for all comparisons < 0.001). Falls was defined as a fall within the last 2 years (HRS) or in the last 3 months (jail-based population); incontinence defined as a response of “yes” to “have you lost any amount of urine beyond your control?” in the last 2 years (HRS) or the last 3 months (jail-based population); Functional impairment was defined as difficulty in one or more of the five Activities of Daily Living; mobility impairment defined as difficulty walking several blocks; hearing impairment defined as a response of “poor” or “fair” to “Is your hearing excellent, very good, good, fair or poor?”; multimorbidity defined as two or more medical conditions (hypertension, diabetes, cancer, lung disease, heart disease, stroke, arthritis), including HIV and Hepatitis C for jail inmates

In age-adjusted analyses, each geriatric condition was more common in jail-based participants than in HRS participants overall and in lowest net worth quintile HRS participants, Fig. 1. Jail-based participants reported significantly higher rates of functional impairment (34% vs. 10% HRS overall vs. 19% HRS lowest quintile of net worth, *p* < 0.001 for both comparisons), mobility impairment (42% vs. 18% vs. 31%, *p* < 0.001 for both comparisons), and hearing impairment (45% vs. 21% vs. 29%, *p* < 0.001 for both comparisons). Compared to both the overall HRS and lowest quintile of net worth populations, jail inmates also had higher rates of multimorbidity, (69% vs. 46% vs. 56%, *p* < 0.001 for both comparisons). Jail-based participants also had significantly higher rates of urinary incontinence (27% vs. 9% vs 11%, *p* < 0.001 for both comparisons) and falls (30% vs. 22% vs. 23%, *p* < 0.001 for both comparisons) than both HRS groups.

### Prevalence of geriatric conditions according to age

Rates of falls and multimorbidity among older jail inmates were statistically similar to rates found among those age 65 to 69 in the overall HRS population (for falls, 30% among older jail inmates versus 28% among the overall HRS population age 65 to 69, *p* = 0.48; for multimorbidity, 69% vs. 67%, *p* = 0.47), Table 2. Rates of each of the other four geriatric conditions were statistically similar to HRS age groups in their 70s and 80s. A similar age gap persisted when comparing jail-based participants to HRS participants in the lowest net worth quintile, though the age gap diminished somewhat for mobility impairment, hearing impairment, functional impairment, and incontinence, Table 3.

**Table 2:** Older adults in jail experienced geriatric conditions at rates statistically similar to those in community-dwelling older adults participating in the Health and Retirement Study (HRS) in their 60s and 70s

Geriatric Condition	% in Jail	% in overall HRS population *(p-value)*				
	Mean Age = 59.4	Age 65–69	Age 70–74	Age 75–79	Age 80–84	Age 85+
Functional Impairment^a^	34%	10%	13%	15%	22%	26%
		(< 0.001)	(< 0.001)	(< 0.001)	(< 0.001)	(0.040)
Mobility Impairment^b^	42%	22%	31%	32%	41%	50%
		(< 0.001)	(< 0.001)	(0.006)	(0.71)	(0.057)
Recent Fall^c^	30%	28%	31%	32%	41%	48%
		(0.479)	(0.704)	(0.591)	(0.003)	(< 0.001)
Hearing Impairment^d^	45%	27%	27%	34%	35%	43%
		(< 0.001)	(< 0.001)	(0.002)	(0.012)	(0.695)
Incontinence^e^	27%	12%	15%	23%	21%	29%
		(< 0.001)	(< 0.001)	(0.238)	(0.055)	(0.677)
Multimorbidity^f^	69%	67%	71%	79%	84%	84%
		(0.471)	(0.584)	(0.003)	(< 0.001)	(< 0.001)

^a^Difficulty in one or more of the five Activities of Daily Living (bathing, eating, toileting, dressing, transferring)

^b^Difficulty walking several blocks

^c^A fall within the last 2 years (HRS); A fall within the last 3 months (jail-based population)

^d^A response of “poor” or “fair” to “Is your hearing excellent, very good, good, fair or poor?”

^e^A response of “yes” to “have you lost any amount of urine beyond your control?” in the last 2 years in the HRS population or the last 3 months in the jail-based population

^f^Two or more medical conditions (hypertension, diabetes, cancer, lung disease, heart disease, stroke, arthritis); for jail inmates, HIV and Hepatitis C were also included

**Table 3:** Older adults in jail experienced geriatric conditions at rates statistically similar to those in community-dwelling older adults in the lowest net worth quintile participating in the Health and Retirement Study (HRS) in their 60s and 70s

Geriatric Condition	% in Jail	% in lowest net worth quintile HRS population *(p-value)*				
	Mean Age = 59.4	Age 65–69	Age 70–74	Age 75–79	Age 80–84	Age 85+
Functional Impairment^a^	34%	14%	23%	25%	24%	29%
		(< 0.001)	(0.009)	(0.042)	(0.061)	(0.380)
Mobility Impairment^b^	42%	41%	39%	46%	47%	58%
		(0.845)	(0.492)	(0.433)	(0.414)	(0.096)
Recent Fall^c^	30%	29%	34%	31%	45%	43%
		(0.807)	(0.466)	(0.815)	(0.006)	(0.054)
Hearing Impairment^d^	45%	31%	31%	38%	40%	27%
		(0.008)	(0.011)	(0.198)	(0.489)	(0.017)
Incontinence^e^	27%	18%	23%	19%	21%	32%
		(0.042)	(0.327)	(0.128)	(0.303)	(0.510)
Multimorbidity^f^	69%	79%	74%	80%	81%	78%
		(0.043)	(0.345)	(0.033)	(0.093)	(0.232)

^a^Difficulty in one or more of the five Activities of Daily Living (bathing, eating, toileting, dressing, transferring)

^b^Difficulty walking several blocks

^c^A fall within the last 2 years (HRS); A fall within the last 3 months (jail-based population)

^d^A response of “poor” or “fair” to “Is your hearing excellent, very good, good, fair or poor?”

^e^A response of “yes” to “have you lost any amount of urine beyond your control?” in the last 2 years in the HRS population or the last 3 months in the jail-based population

^f^Two or more medical conditions (hypertension, diabetes, cancer, lung disease, heart disease, stroke, arthritis); for jail inmates, HIV and Hepatitis C were also included

## Discussion

In this study, we found that older adults in jail had a higher burden of many chronic health and geriatric conditions than community-dwelling older adults of similar age. Among older adults in jail with an average age of 59, the prevalence of several geriatric conditions was similar to that found among community dwelling adults age 75 or older. While much of the differential burden of chronic health conditions resolved when jail-based participants were compared to community-dwelling adults in the lowest quintile of net worth (<$24,000), the higher rates of geriatric conditions persisted. The high rates of geriatric conditions found at younger ages in this population suggest that services directed towards assessment and management of geriatric conditions are needed in jails and that these services should be targeted to older adults at ages younger than would be the norm in the community.

This is the first study, to our knowledge, to directly compare the prevalence of geriatric conditions among older adults in jail to those in non-incarcerated older adults. One study from1992 conducted in Iowa found lower rates of geriatric conditions than were found in this study (Colsher et al. 1992). However, the Iowa study assessed dependence in self-care while this study assessed difficulty with Activities of Daily Living, a more standard assessment of functional impairment in geriatric medicine. Additionally, the majority of participants in the Iowa study were white, and studies show that many geriatric conditions are more common in African-American older adults (Cigolle et al. 2007).

There are many factors that could explain the relatively high rates of geriatric conditions found in older jail inmates in this study. In the community, non-white race/ethnicity is associated with higher rates of geriatric conditions (National Research Council (US) Panel on Race, Ethnicity, and Health in Later Life, et al. 2004). Additional explanatory factors might include current homelessness and/or HIV, both of which are associated with a high burden of geriatric conditions (Brown et al. 2012; Greene et al. 2015). While future studies might consider exploring the explanatory mechanisms leading to a higher burden of geriatric conditions, our descriptive findings suggest that systems of care that have been designed for low-income community-dwelling older adults (such as the Grace Model (Counsell et al. 2006)) will likely need to be adapted further to meet the needs of older adults in jail.

Our findings provide more epidemiologic evidence of so-called “accelerated aging” in older jail inmates. This finding is important because it buttresses the argument that incarcerated persons should be considered to be of “older” age when they are in their 50s and that a geriatrics-based approach to care is appropriate for relatively young persons in correctional facilities (Williams et al. 2012b). While this study took place in jail, it is likely that older adults in prison also experience disproportionately high rates of premature geriatric conditions since they also experience a high burden of chronic medical conditions, disability and stressors across the lifespan (Maschi et al. 2011; Binswanger et al. 2009; Williams et al. 2006; Williams et al. 2009). Future studies should investigate this relationship to determine the extent to which the phenomenon of clinically meaningful premature aging exists across criminal justice populations.

Jail- and community-based healthcare systems are increasingly faced with the challenge of providing appropriate and effective care to the growing number of socioeconomically vulnerable older adults who cycle through jail. Incarcerated older adults generate up to 9 times the cost of younger prisoners while in custody (Ahalt et al. 2013) and many older adults cycling through jail rely on costly emergency department services while in the community (Chodos et al. 2014; Humphreys et al. 2017). Thus addressing the needs of older adults while incarcerated and upon community re-entry is critical (Maschi et al. 2013). Clinical care models have been developed to provide cost-efficient, culturally appropriate care to low-income older patients (Coleman et al. 2006; Counsell et al. 2007). These models take a holistic approach to care, prioritizing assessment and management of geriatric conditions including functional ability (e.g., difficulty performing Activities of Daily Living - bathing, dressing, eating, transferring and toileting), incontinence, frequent falls, and multimorbidity while simultaneously tending to patients’ psychosocial needs. This approach has proven successful (Coleman et al. 2006; Counsell JAMA 2007) in part because geriatric conditions have a greater impact on the overall older adult health than do individual medical conditions (Lee et al. 2008; Shih et al. 2015). Our findings describing a high burden of geriatric conditions – and a relatively younger age of onset of these conditions – among older adults in the criminal justice system suggests opportunities to adapt these care models to better meet the needs of this particularly vulnerable population, for example by accounting for high rates of homelessness, substance use disorders, and extreme poverty – all factors not included in the HRS. In addition, because African-American older adults were significantly over-represented in the jail population, care models should also ensure culturally competent care.

Several limitations should be considered when evaluating our results. First, the study does not include women. Incarcerated women experience even higher rates of chronic disease than men (Binswanger et al. 2010) suggesting that this study’s findings may underestimate the burden of illness present in incarcerated older women. This study’s jail-based participants were enrolled after having been in jail for at least 48 h and via consecutive sampling. The 48-h timeframe was chosen to minimize the burden to participants who are busy travelling between jail and court in the first hours after their arrival at jail, and because it more accurately describes the population of older adults who will require jail-based healthcare services rather than simply acute care triage in the first hours of detainment. A consecutive sampling approach was chosen because it can minimize selection biases such as volunteerism and can help account for seasonal variations (such as fewer arrests over holidays) (Hulley et al. 2007). Future studies might consider alternate strategies to enroll participants earlier in the course of detainment. In addition, while this study was limited to one urban jail, the predominance of non-white participants living below the federal poverty line suggests demographic similarities to other jail systems. Additionally, the San Francisco jail, by virtue of being in a city with universal access to healthcare (through Healthy San Francisco) (Finley and Brigham 2010) could represent a healthier population than other jail systems meaning that this study may actually overestimate the health of older adults in jail nationwide.

The jail-based participants in this study were found to experience high rates of Hepatitis C, HIV, and homelessness – all measures that are not collected by HRS. This study underscores the growing need for national studies about aging-related health to include measures of HIV, Hepatitis C, and a host of behavioral health and social factors such as homelessness, substance use disorders, and extreme poverty. Studies to better understand mechanisms over the life course that contribute to the differences in health found here are also needed to inform interventions in medically vulnerable young and middle-aged adults that might reduce later life health disparities. Finally, although nearly all of the measures collected were identical in the two studies, a shorter reporting period for falls and urinary incontinence in the jail study may mean that the prevalence of these geriatric conditions is underestimated in the jail-based study participants.

## Conclusions

A rapidly growing population of older adults cycle in and out of U.S. jails; many face multiple chronic conditions and use the emergency department with frequency when they are not in jail (Chodos et al. 2014; Humphreys et al. 2017; Bolano et al. 2016). This is the first study to directly compare geriatric conditions among older adults in jail to those in the community. Our findings suggest that an earlier age of eligibility for geriatric assessment and services in jails is warranted, that geriatrics-based care models adapted for use in the jail setting are needed, and that geriatric expertise among clinicians practicing in correctional settings is increasingly critical. Existing geriatric care models for low-income, diverse community-dwelling older adults should be adapted to meet the complex medical, social and behavioral health needs of the rapidly growing population of “younger-old” who interact with community criminal justice systems.

## References

[CR1] Aday RH (2003). Aging prisoners: Crisis in American corrections.

[CR2] Ahalt C, Sudore R, Bolano M, Metzger L, Darby AM, Williams B (2017). "teach-to-goal" to better assess informed consent comprehension among incarcerated clinical research participants. AMA Journal of Ethics.

[CR3] Ahalt C, Trestman RL, Rich JD, Greifinger RB, Williams BA (2013). Paying the price: The pressing need for quality, cost, and outcomes data to improve correctional health care for older prisoners. Journal of the American Geriatrics Society.

[CR4] Beck, AJ, & Berzofsky, M (2010). *Sexual Victimization in Prison and Jails Reported by Inmates, 2008–2009 [Table 6]. NCJ 231169*, (p. 91). Washington (DC): Department of Justice. Office of Justice Programs, Bureau of Justice Statistics.

[CR5] Bedard, R, Metzger, L, Williams, B. (2017). Ageing prisoners: An introduction to geriatric health-care challenges in correctional facilities. *International Review of the Red Cross*, 1–23. 10.1017/S1816383117000364.

[CR6] Binswanger, IA, Krueger, PM, Steiner, JF. (2009). Prevalence of chronic medical conditions among jail and prison inmates in the USA compared with the general population. *Journal of Epidemiology and Community Health*, *63*(11), 912–919. 10.1136/jech.2009.090662 19648129

[CR7] Binswanger IA, Merrill JO, Krueger PM, White MC, Booth RE, Elmore JG (2010). Gender differences in chronic medical, psychiatric, and substance-dependence disorders among jail inmates. American Journal of Public Health.

[CR8] Binswanger IA, Stern MF, Deyo RA (2007). Release from prison--a high risk of death for former inmates. The New England Journal of Medicine.

[CR9] Bolano M, Ahalt C, Ritchie C, Stijacic-Cenzer I, Williams B (2016). Detained and distressed: Persistent distressing symptoms in a population of older jail inmates. Journal of the American Geriatrics Society.

[CR10] Brown RT, Kiely DK, Bharel M, Mitchell SL (2012). Geriatric syndromes in older homeless adults. Journal of General Internal Medicine.

[CR11] Bush K, Kivlahan DR, McDonell MB, Fihn SD, Bradley KA (1998). The AUDIT alcohol consumption questions (AUDIT-C): An effective brief screening test for problem drinking. Ambulatory care quality improvement project (ACQUIP) Alcohol Use Disorders Identification Test. Archives of Internal Medicine.

[CR12] Bush TL, Miller SR, Golden AL, Hale WE (1989). Self-report and medical record report agreement of selected medical conditions in the elderly. American Journal of Public Health.

[CR13] Chodos AH, Ahalt C, Cenzer IS, Myers J, Goldenson J, Williams BA (2014). Older jail inmates and community acute care use. American Journal of Public Health.

[CR14] Cigolle CT, Langa KM, Kabeto MU, Tian Z, Blaum CS (2007). Geriatric conditions and disability: The health and retirement study. Annals of Internal Medicine.

[CR15] Coleman EA, Parry C, Chalmers S, Min SJ (2006). The care transitions intervention: Results of a randomized controlled trial. Archives of Internal Medicine.

[CR16] Colsher PL, Wallace RB, Loeffelholz PL, Health SM (1992). Status of older male prisoners: A comprehensive survey. American Journal of Public Health.

[CR17] Counsell SR, Callahan CM, Buttar AB, Clark DO, Frank KI (2006). Geriatric resources for assessment and Care of Elders (GRACE): A new model of primary care for low-income seniors. Journal of the American Geriatrics Society.

[CR18] Counsell SR, Callahan CM, Clark DO (2007). Geriatric care management for low-income seniors: A randomized controlled trial. JAMA.

[CR19] Covinsky KE, Justice AC, Rosenthal GE, Palmer RM, Landefeld CS (1997). Measuring prognosis and case mix in hospitalized elders. The importance of functional status. Journal of General Internal Medicine.

[CR20] Covinsky KE, Palmer RM, Counsell SR, Pine ZM, Walter LC, Chren MM (2000). Functional status before hospitalization in acutely ill older adults: Validity and clinical importance of retrospective reports. Journal of the American Geriatrics Society.

[CR21] Darrell G, Beck A (1997). Prison and Jail Inmates at Midyear 1996. NCJ 162843.

[CR22] Finley D, Brigham T (2010). Creating a network of care: Healthy San Francisco connects uninsured residents to a primary care home. Healthcare Executive.

[CR23] Greene M, Covinsky KE, Valcour V (2015). Geriatric syndromes in older HIV-infected adults. Journal of acquired immune deficiency syndromes (1999).

[CR24] Hanson RK, Letourneau EJ, Olver ME, Wilson RJ, Miner MH (2012). Incentives for offender research participation are both ethical and practical. Criminal Justice and Behavior.

[CR25] Harris PA, Taylor R, Thielke R, Payne J, Gonzalez N, Research CJG (2009). Electronic data capture (REDCap)--a metadata-driven methodology and workflow process for providing translational research informatics support. Journal of Biomedical Informatics.

[CR26] Hulley SB, Cummings SR, Browner WS, Grady DG, Newman TB (2007). Designing clinical research, third edition.

[CR27] Humphreys, J, Ahalt, C, Stijacic-Cenzer, I, Widera, E, Williams, B. (2017). Six-month emergency department use among older adults following jail incarceration. *Journal of Urban Health*. [Epub ahead of print] 10.1007/s11524-017-0208-4 PMC6095758 29204845

[CR28] Hwang SW, Chambers C, Katic M (2016). Accuracy of self-reported health care use in a population-based sample of homeless adults. Health Services Research.

[CR29] James DJ, Glaze LE (2006). Mental health problems of prison and jail inmates. NCJ 213600.

[CR30] Juster F (1995). An overview of the health and retirement study. Journal of Human Resources: Special Issue on the Health and Retirement Study: Data Quality and Early Results.

[CR31] Lee SJ, Go AS, Lindquist K, Bertenthal D, Covinsky KE (2008). Chronic conditions and mortality among the oldest old. American Journal of Public Health.

[CR32] Marquez, M. (2011). Homeless emergency assistance and rapid transition to housing (HEARTH): Defining “homeless” final rule. *U.S. Department of Housing and Urban Developmen* https://www.hudexchange.info/resources/documents/HEARTH_HomelessDefinition_FinalRule.pdf.Accessed 11 April, 2016.

[CR33] Maschi T, Gibson S, Zgoba KM, Morgen K (2011). Trauma and life event stressors among young and older adult prisoners. Journal of Correctional Health Care.

[CR34] Maschi T, Morrisey MB, Leigey M (2013). The case for human agency, well-being, and community reintegration for people aging in prison: A statewide case analysis. Journal of Correctional Health Care.

[CR35] Minton T, Zeng Z (2015). Jail Inmates at Midyear 2014. NCJ 248629..

[CR36] Anderson NB, Bulatao RA, Cohen B, National Research Council (US) Panel on Race, Ethnicity, and Health in Later Life (2004). Critical Perspectives on Racial and Ethnic Differences in Health in Late Life.

[CR37] Pollack CE, Chideya S, Cubbin C, Williams B, Dekker M, Braveman P (2007). Should health studies measure wealth? A systematic review. American Journal of Preventive Medicine.

[CR38] Psick Z, Simon J, Brown R, Ahalt C (2017). Older and incarcerated: Policy implications of aging prison populations. International Journal of Prisoner Health.

[CR39] Shih SL, Gerrard P, Goldstein R (2015). Functional status outperforms comorbidities in predicting acute care readmissions in medically complex patients. Journal of General Internal Medicine.

[CR40] Skinner HA (1982). The drug abuse screening test. Addictive Behaviors.

[CR41] Snyder HN (2012). Arrest in the United States, 1990–2010. NCJ 239423.

[CR42] StataCorp. (2011). *Stata Statistical Software*: Release 12. College Station, TX: StataCorp LP.

[CR43] Sudore RL, Landefeld CS, Williams BA, Barnes DE, Lindquist K, Schillinger D (2006). Use of a modified informed consent process among vulnerable patients: A descriptive study. Journal of General Internal Medicine.

[CR44] Walmsley, R (2000-2011). *World prison population list*. London: International Centre for Prison Studies Available from: http://www.icpr.org.uk/media/41356/world_prison_population_list_11th_edition.pdf.

[CR45] Ware J, Kosinski M, Keller SD (1996). A 12-item short-form health survey: Construction of scales and preliminary tests of reliability and validity. Medical Care.

[CR46] Williams BA, Baillargeon JG, Lindquist K (2009). Medication prescribing practices for older prisoners in the Texas prison system. American Journal of Public Health.

[CR47] Williams BA, Goodwin JS, Baillargeon J, Ahalt C, Walter LC (2012). Addressing the aging crisis in U.S. criminal justice health care. Journal of the American Geriatrics Society.

[CR48] Williams BA, Lindquist K, Sudore RL, Strupp HM, Willmott DJ, Walter LC (2006). Being old and doing time: Functional impairment and adverse experiences of geriatric female prisoners. Journal of the American Geriatrics Society.

[CR49] Williams, BA, Stern, MF, Mellow, J, Safer, M, Greifinger, RB. (2012). Aging in correctional custody: Setting a policy agenda for older prisoner health care. *American Journal of Public Health*, *102*(8), 1475–1481. 10.2105/AJPH.2012.300704 PMC3464842 22698042

